# Identification of Metabolic Pathways Essential for Fitness of *Salmonella* Typhimurium *In Vivo*

**DOI:** 10.1371/journal.pone.0101869

**Published:** 2014-07-03

**Authors:** Lotte Jelsbak, Hassan Hartman, Casper Schroll, Jesper T. Rosenkrantz, Sebastien Lemire, Inke Wallrodt, Line E. Thomsen, Mark Poolman, Mogens Kilstrup, Peter R. Jensen, John E. Olsen

**Affiliations:** 1 Department of Veterinary Disease Biology, Faculty of Health and Medical Sciences, University of Copenhagen, Frederiksberg, Denmark; 2 Department of Medical and Biological Sciences, Faculty of Health and Life Science, Oxford Brookes University, Oxford, United Kingdom; 3 Center for Systems Microbiology, Department of Systems Biology, Technical University of Denmark, Kongens Lyngby, Denmark; Institut National de la Recherche Agronomique, France

## Abstract

Bacterial infections remain a threat to human and animal health worldwide, and there is an urgent need to find novel targets for intervention. In the current study we used a computer model of the metabolic network of *Salmonella enterica* serovar Typhimurium and identified pairs of reactions (cut sets) predicted to be required for growth *in vivo.* We termed such cut sets synthetic auxotrophic pairs. We tested whether these would reveal possible combined targets for new antibiotics by analyzing the performance of selected single and double mutants in systemic mouse infections. One hundred and two cut sets were identified. Sixty-three of these included only pathways encoded by fully annotated genes, and from this sub-set we selected five cut sets involved in amino acid or polyamine biosynthesis. One cut set (*asnA/asnB*) demonstrated redundancy *in vitro* and *in vivo* and showed that asparagine is essential for *S.* Typhimurium during infection. *trpB/trpA* as well as single mutants were attenuated for growth *in vitro*, while only the double mutant was a cut set *in vivo,* underlining previous observations that tryptophan is essential for successful outcome of infection. *speB/speF,speC* was not affected *in vitro* but was attenuated during infection showing that polyamines are essential for virulence apparently in a growth independent manner. The *serA/glyA* cut-set was found to be growth attenuated as predicted by the model. However, not only the double mutant, but also the *glyA* mutant, were found to be attenuated for virulence. This adds glycine production or conversion of glycine to THF to the list of essential reactions during infection. One pair (*thrC/kbl*) showed true redundancy *in vitro* but not *in vivo* demonstrating that threonine is available to the bacterium during infection. These data add to the existing knowledge of available nutrients in the intra-host environment, and have identified possible new targets for antibiotics.

## Introduction

Despite substantial progress in infection biology research over the last decades, bacterial infections remain a major threat to human and animal health worldwide. With the rapid evolution and spread of antimicrobial resistance [1], [2], development of new strategies to fight bacterial infections is urgently needed.

Bacterial metabolism constitutes a recent area of interest with regard to host-pathogen interactions because of the potential of identifying novel anti-infective targets that inhibits pathogen survival in the host [3]–[6]. However, knowledge about the nutritional environment inside the host has for most pathogens only been scarcely described, and the majority of this knowledge has been inferred from gene expression studies of host-pathogen interactions in cell cultures or investigated through virulence studies of a limited number of specific auxotrophic mutants [7]–[12].

System level approaches describing the metabolic capacities of bacterial pathogens has revealed that bacterial metabolism can be highly robust with extensive redundancies, not only within the pathogen but also between the pathogen and the host [13]–[17]. As a consequence, the identification of new essential metabolic targets for treatment of bacterial infections has been challenging [18]. To meet this challenge it has been proposed to target reactions in redundant pathways constituting cut sets [19], i.e. sets of metabolic reactions that when removed from the organism in conjunction proves detrimental, while each of them are dispensable. Cut sets could be of any size, but for development of antimicrobial agents, cut sets above two reactions are impractical. In the case where a pair of redundant reactions can be removed from the network by deleting exactly two genes, these genes are referred to as a synthetic lethal pair when they cause absolute absence of growth [20]. Since we identify pairs of reactions that cause inability to grow in defined minimal medium, we have coin the term “synthetic auxotrophic” and use that to describe the situation where the model predicts no growth when exactly two genes are removed.

Exhaustive identification of synthetically auxotrophic pairs using purely experimental approaches would involve constructing and characterizing many thousands of mutant strains, which is, if not infeasible, cumbersome. Also, since this approach would be limited to combinations of two genes, possible combinations of pairs of enzymes encoded by redundant genes would be missed. An alternative, explored here, is to use a computer model of the metabolic network and identify pairs of reactions that when removed from the network renders it incapable of synthesizing biomass, i.e. cut sets. Here we investigate the metabolic network of *Salmonella enterica* serovar Typhimurium (*S*. Typhimurium) using a published genome-scale metabolic model (GSM) [21]. GSMs are models involving all metabolic reactions available to an organism, ultimately encoded by its genome. GSMs published today are stoichiometric models, owing to the lack of system-wide kinetic data for most metabolic systems [22], [23]. The only knowledge required for construction of stoichiometric models is the structure of the network, i.e. the reactions involved and their stoichiometry. Construction and analysis of metabolic models for bacteria have been reviewed by Durot *et al.* [24].

In the current study we have combined *in silico* metabolic modeling to identify predicted redundancies in pathways in the metabolism with a targeted validation by infection experiments with selected synthetic auxotrophic pairs involved in amino acid and polyamine biosynthesis. Specifically, we have used *S.* Typhimurium as a model to investigate the role of 5 cut sets, suggested by computational analysis. Results have disclosed specific information about the available nutrients in the host environment, and we have identified potential novel combined targets for antimicrobial therapy.

## Methods

### Bacterial strains and growth conditions

*S.* Typhimurium 4/74 was used as wild-type strain in all experiments. This strain has been described previously and its virulence is well defined [25]. The restriction deficient strain *S.* Typhimurium KP1274 was used as primary recipient for plasmids [26]. *S.* Typhimurium strains were maintained in LB media (Oxoid). For solid medium, 1.5% agar was added to give LB agar plates. Chloramphenicol (10 µg ml^−1^), kanamycin (50 µg ml^−1^) or carbenicillin (75 µg ml^−1^) was added as required. Growth phenotypes were investigated in LB (Difco), M9 minimal media (2 mM MgSO_4_, 0.1 mM CaCl_2_, 0.4% glucose, 8.5 mM NaCl, 42 mM Na_2_HPO_4_, 22 mM KH_2_PO_4_, 18.6 mM NH_4_Cl) or M9 minimal media supplemented with 50 mg/l alanine, thiamine, glycine, glutamine, proline, arginine, aspartate, glutamate (M9+) as suggested [27]. *Escherichia coli* Top10 competent cells were used for DNA cloning and were grown in LB media or on LB agar plates at 37°C with appropriate selection.

### Construction of strains and plasmids

Gene deletions and concomitant insertions of an antibiotic resistance cassette were constructed using Lambda Red mediated recombination as described elsewhere [28]. All constructs were verified by PCR and moved into a clean background via P22 phage transduction as previously described [29]. Double mutant strains were also constructed by P22-mediated transductions. Primers used to construct mutants are listed in Table S1. The genotype of mutants constructed appear in Table 1. Genetic complementation of mutant in *asnA/asnB* was achieved by cloning *asnA* into pSLD64, while genetic complementation of other genes was achieved by PCR amplification and subsequent cloning of genes into pACYC177, essentially as described [29]. Primers used are listed in Table S1. Restriction enzymes used for cloning were *Xho*I and *Bam*HI. Enzymes were used according to the manufacturer’s recommendentation (Thermo Scientific). All constructs were verified by restriction analysis and sequencing.

**Table 1:** In vitro and in vivo phenotypes of cut sets.

Synthetic auxotrophic pair#	Mutant genotype¤	Growth$			Competitive index (CI)¥
		LB	M9	M9+£	
(72)^a^ ASNSYNB-RXN, ASNSYNA-RXN	ΔSTM3877 (*asnA*)	+	+	+	1.33+/−0.53
	ΔSTM0680 (*asnB*)	+	+	+	0.61+/−0.29
	ΔSTM0680/STM3877	+	-	-	0.05+/−0.009*
	Δ*asnA*/*asnB+*pSLD64-*asnA*	nd	+	+	0.33+/−0.18**
(2) PGLYCDEHYDROG-RXN,GLYOHMETRANS-RXN	ΔSTM3062 (*serA*)	+	-	+	1.02+/−0.15
	ΔSTM2555 (*glyA*)	+	-	+	0.02+/−0.02*
	ΔSTM3062/STM2555	+	-	-	0.01+/−0.01*
	*serA/glyA* +pACYC177-*glyA*	nd	nd	nd	0.5+/−0.17**
(14) THRESYN-RXN, AKBLIG-RXN	ΔSTM0004 (*thrC*)	+	(+)	(+)	0.87+/−0.18
	ΔSTM3709 (*kbl*)	+	+	+	0.61+/−0.31
	ΔSTM0004/STM3709	+	-	-	0.88+/−0.22
(11) AGMATIN-RXN, ORNDECARBOX-RXN	ΔSTM3078 (*speB*)	+	+	+	0.37+/−0.15
	ΔSTM0701 (*speF*)/STM3114 (s*peC*)	+	+	+	1.26+/−0.53
	ΔSTM3078/STM07017+STM3114	+	+	+	0.0+/−0
(36) TRYPSYN-RXN	ΔSTM1727 (*trpA*)	+	-	-	1.27+/−0.0
	ΔSTM1727+pACYC1727-*trpA*	+	+	nd	nd
	ΔSTM1726 (*trpB*)	+	-	-	1.44+/−0.23
	ΔSTM1726+pACYC1726-*trpB*	+	(+)	nd	nd
	ΔSTM1726-STM1727	+	-	-	0.31+/−0.11
	Δ*trpA/trpB* + pACYC1727-*trpA*		-	nd	nd

nd: Not done;

# Reaction names is according to MetaCyc database [33].

¤ LT2 gene numbers.

$ Growth phenotype is indicated as: +: growth as wild type strain, (+): growth observed but with rates below wild type strain, -: no growth observed.

£ The media used as input in the model analysis and whether model prediction was considered true depended on the growth phenotype in this medium.

¥ C.I.: competitive index of 1.00 corresponds to Wild type virulence.

# Prediction number in Table S2.

*Significantly different from WT strains.

**Significantly different from mutated strain without complementation.

### Mouse mixed infections

Female C57/BL6 mice (Nramp-) (20–25 g) were used to assess *in vivo* fitness of bacterial strains as previously described [29]. Briefly, mice were inoculated I.P. with 0.1 ml of a 50∶50 mixture of wild type and mutated bacteria suspended in physiological saline. To prepare the inoculum, strains were grown for 16 hrs, 200 rpm at 37°C in LB media. Equal amounts of wild type and mutated strains were mixed before the infection to give a challenge dose of 5×10^3^ bacteria of each strain. The exact c.f.u. and ratio between wild type and mutated strains were enumerated by plating as described below. Mice were killed at 6 days post-inoculation by cervical dislocation. Severely affected animals were sacrificed early to this time point for animal welfare reasons, but otherwise treated as the rest of the group. The spleens were removed aseptically and bacteria recovered and enumerated after plating a dilution series on to LB agar. One hundred colonies were randomly picked and tested for resistance to the relevant antibiotic to determine the proportion of mutant strains. The competitive index was calculated as the mutant/wild type ratio of the output versus the mutant/wild type ratio of the inoculum.

### Ethical statement

Mice experiments were conducted with permission from the Danish Animal Experiments Inspectorate, license number: 2009/561–1675.

### Statistical analysis

Statistical significance of the differences between wild type and mutant strains was determined using GraphPad Prism version 5.0 (GraphPad software) with one-sample t-test analysis. Grubb’s outlier test was performed to exclude outliers with a significance of 0.05.

### Simulation studies using a metabolic model

The model used, which contains 1099 reactions, has been described elsewhere [21]. All model analysis was conducted assuming a medium consisting of M9 added a number of non-essential amino acids as detailed under growth experiments above.

The model was analyzed using Flux Balance Analysis [30], [31], as described previously [21]. The Linear Program (LP) was set to minimize the flux-sum of the network, subject to steady-state assumption, and the constraint that all biomass components must be produced with biologically realistic rates, as stated in equation 1.
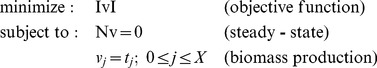
(1)where |v| is the absolute value of the flux vector, Nv = 0 expresses the steady-state assumption, and vector t contains the rates of export of biomass components from the network.

All model analysis was carried out using the ScrumPy package [32]. Identification of cut sets reactions was done in two steps – (*i*) all cut sets of size one were identified by attempting to solve equation 1 with each reaction sequentially forced to carry zero flux (except the trivial set involving all reactions in vector **t**); (*ii*) all two-reaction combinations which were not supersets of reactions identified in (*i*) were sequentially set to zero and combinations which rendered the problem infeasible were recorded. For each cut set the cause of the lethality was analyzed by identifying which biomass components were not produced when the pair was removed from the network. This was done by attempting to solve the FBA (with a given pair fixed to zero flux) for each component of vector **t** fixed to a non-zero value, whilst fixing all other components to zero.

The gene associations of the selected reaction pairs were obtained from the BioCyc database [33], through the PyoCyc functionality of the ScrumPy package, as described in Hartman *et al.* [21].

## Results and Discussion

*S.* Typhimurium is an important intracellular pathogen of both humans and animals causing self-limiting enteritis and life threatening systemic infections. Replication and dissemination of *S.* Typhimurium inside the host is essential for establishing a systemic infection and is dependent on the ability to acquire and utilize the nutrients available in this niche in addition to the ability to synthesize essential nutrients that are only present in limited concentrations [33]. *S.* Typhimurium causes a systemic disease in susceptible mice, thus serving as a suitable model for studying bacterial systemic infections [34].

### Computational cut set analysis

A metabolic model of *S.* Typhimurium [21] was used to detect cut sets of size two. The range of nutrients available to *S.* Typhimurium at different points during the systemic stage of infection is largely unknown, but contains glucose and certain amino acids [35]. Recent studies have revealed that also other carbohydrates constitute a suitable nutrient for *S.* Typhimurium during infection [36]. The robustness of a metabolic model is determined by the complexity of the media components assumed to be available to the network, i.e. the number of external metabolites, and the degree of redundancy of the network. *S.* Typhimurium exhibits carbon catabolite repression [37], i.e. it shows preference for glucose over other carbon sources. Still the use of other carbon sources would change network performance, because it will differ where the other carbon sources go in and metabolites go out. However, block of the glucose catabolism, as investigated in the current study, would also interfere with the catabolism of other possible sources. Since the focus here was on identification of cut sets, which were to be confirmed by biological experimentation, we used the pragmatic approach of assuming a medium of minimal, but realistic complexity with glucose as the carbon source.

**Figure 1:**
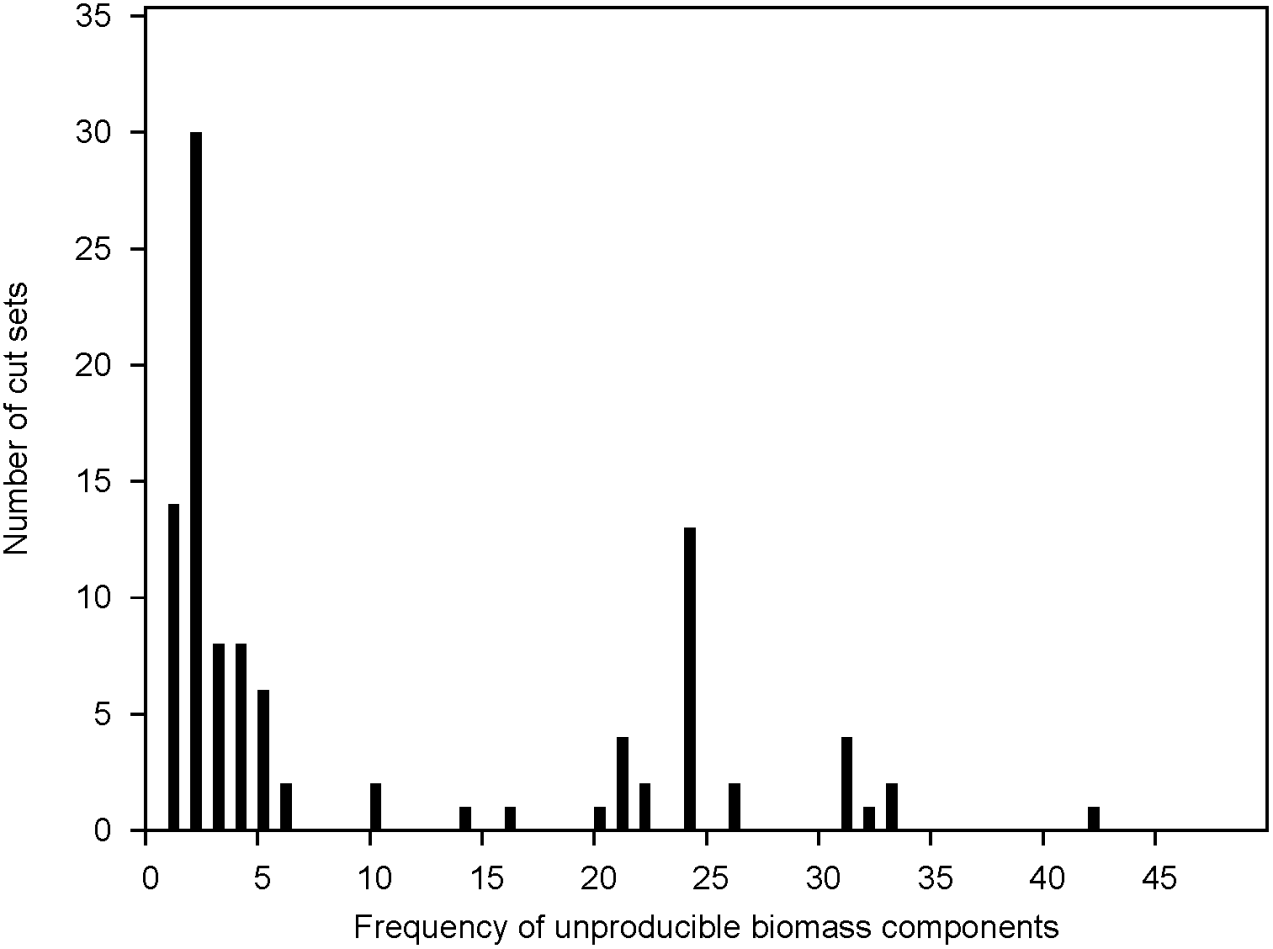
Frequency of biomass components predicted by the model to be affected by the 102 cut sets identified.

The damage analysis resulted in a list of 102 reaction pairs hampering the production of essential biomass components (Table S2). The number of biomass components that were not produced with a given cut set varied from one to all, with the distribution shown in Figure 1. As can be seen in the figure, few cut sets had the results that many components were not produced, while the majority affected only few components. Two groups of cut sets deviated from the general pattern. They were those that prevented synthesis of two components and 24 components. The first group was dominated by cut sets abolishing spermidine and putrescine biosynthesis and abolished amino acid biosynthesis was also prominent in this group. The second group involved predominantly various combinations of reactions from the Entner-Doudoroff and pentose phosphate pathway. Interestingly, genetically redundant genes encode many of these highly detrimental cut sets, and most sets in this group would require 5–6 gene deletions in order to abolish their function. The initial 102 cut sets were reduced to 63 by removing all sets where any reaction lacked gene annotation.

A number of genes were predicted by the analysis to be essential (i.e. they formed a cut-set of two reaction pairs where the same gene encoded both reactions or they formed a cut-set together with the (artificial) transporter reaction of a molecule synthesized by the reaction (see Table S2). Five of these were selected to investigate the preciseness of predictions, since literature studies are available for essential genes in *S.* Typhimurium during systemic mouse infection [38]: *prsA, argD, fbp, adk* and *nadE*.

The homolog of STM1780 (*prsA*) has been reported to be conditional essential in *E. coli* [39], an viable *E. coli* null mutants require additional mutations and several additional metabolites to be viable [40]. In a global analysis of the importance of genes for systemic *Salmonella* infection, *prsA* was not reported, suggesting that *prsA* mutants are not viable [38]. Since this was the first gene to be considered, we chose to confirm this observation. In support of the model prediction, we could not produce a mutant in this gene, despite several attempts, suggesting that the gene is essential in *S.* Typhimurium under the conditions used for construction of mutants.

*argD* encodes a bi-functional enzyme in arginine and lysine biosynthesis [33]. This gene too, was not hit by Chadhuri *et al.* [38] in their random mutation analysis, suggesting that it is essential and cannot be analysed for virulence impact by mutational analysis. *fbp* encodes a fructose-1, 6,bi-phosphatase, which participates both in glycolysis and glyconeogenesis [33]. A functional glycolysis pathway has been shown to be essential for survival inside macrophages as well as for successful mouse infection [3], and thus *fbp* must be considered essential for *in vivo* fitness. The adelynate kinase encoded by *adk* participates in purine and pyrimidine metabolism as well as adenoside nucleotide *de novo* biosynthesis [33]. Strains with point mutations in the promoter or coding region of this gene showed altered osmo-protection phenotype [40], but to the best of our knowledge, no investigation of the role in virulence has been performed. As for other genes analysed here, no mutant hits were reported by Chadhuri *et al.* [38]. *nadE* encodes an enzyme responsible for both glutamine dependent and glutamine independent NAD^+^ synthesis [33], and this enzyme was also not in the pool of transposon mutants [38]. Together, literature reports, previous studies of essential genes in *Salmonella* mouse infection [38] and our own study of the gene *prsA* suggested that model predictions were precise and that we could proceed to more cumbersome testing of double lethal pairs.

### Growth validation of model predictions

Among the 63 cut sets identified by the analysis, five cut sets were selected for further analysis (Table 1). The cut sets were picked among those that only involved two or three genes and which abolished amino acid or polyamine biosynthesis. The focus on cut sets with only two or three genes was done for practical reasons, but with a view to future use as possible targets, it is also most realistic to base this on simple combinations.

Genes in the selected reactions were subjected to mutational analysis. Initially we tested growth of the mutants *in vitro*. The growth phenotype was investigated by measuring the growth in liquid rich media (LB), minimal media with glucose (M9) and M9 additionally supplemented with specific amino acids (M9+). The composition of the latter medium corresponded to the input provided in the computational cut set analysis. According to the model analysis, single mutants should grow like the wild type strain, whereas double-mutants were expected to be unable to grow in M9+ as they would be auxotrophic for at least one biomass component. Table 1 lists growth reactions of the cuts sets, while growth performances in M9+ are shown graphically in Figure S1.

Of the pairs tested, three (*asnA/asnB*; *serA/glyA* and *thrC/kbl*) confirmed the cut set analysis since single mutants grew well in M9+, while the double mutant did not grow. Contrary to this, even the double mutant in the pair *speB/speF,speC* was not attenuated for growth, while both single and double mutants of *trpA/trpB* were affected for growth (Table 1 and Figure S1).

### Investigation of attenuation of mutant strains

*In vivo* fitness of both single and double mutants was tested in the mouse model of typhoid fever in competition with the isogenic wild type parent. Bacterial load in the spleen of each mouse was determined and the competitive index (C.I.) was calculated for each infection pair (Table 1). The *in vivo* fitness of a mutant was scored as low if the C.I. was less than 0.33 and/or significantly different from 1.0. Based on these criteria, four double mutants were less fit than the wild type strain (*asnA/asnB*; *serA/glyA*; *trpA/trpB* and *speB/speC,speF*) indicating that these reaction pairs contributed significantly to the fitness of the bacterium during infection, while the double mutant *thrC/kbl* was unaffected. Interestingly the single *glyA* mutant also proved attenuated, despite its ability to grow *in vitro* (Table 1).

### Details of individual reaction pairs

Based on these results, the predictions from the model analysis, which related to growth phenotype in the M9+ media used as input in the model, proved correct for three out of 5 cut sets. However, with a view to identification of new targets for infection control, the model analysis will always be considered an initial screening step, and the *in vivo* testing is the important step. For this part, four out of five predictions gave indications of *in vivo* attenuation.

A clear set of phenotypes came from the analysis of the *asnA* and *asnB* encoded enzymes. Both AsnA and AsnB catalyze the transfer of aspartate to asparagine, although not iso-stoichiometriccally as AsnA uses ammonia as a reactant, where AsnB predominantly uses glutamine [33] (Figure S2). Both of the single mutants, but not the double mutant could grow in media without asparagine (Table 1). The reduction in the C.I. during I.P. infection of mice showed that the asparagine availability during mouse infection is not sufficient for growth of the double mutant. Complementation of the virulence phenotype was obtained in the presence of a wild type copy of the *asnA* gene on a plasmid (Table 1). Based on these results, drugs targeting AsnA and AsnB constitute a potential new anti-infective treatment approach.

A serine auxotroph in *E. coli* is able to grow with glycine as sole precursor for serine synthesis. It was concluded that the breakdown of glycine by the Gcv reaction is sufficient to generate enough methylene-THF to reverse the GlyA reaction, thereby synthesizing serine from glycine [41]. Serine is however also synthesized from the glycolytic intermediate 3-phospho-glycerate (3PG) by the action of the SerA/B/C reactions (Figure S3). In this respect a *serA/glyA* double mutant should form a synthetic auxotrophic couple in the presence of glycine due to serine auxotrophy, while a *serA* or a *glyA* single mutant should grow fine. This was confirmed by growing the mutants in minimal M9+ medium in the presence of glycine (Table 1 and Figure S1). Further, as expected, the *serA* mutant had no infection defects, but interestingly, the *glyA* mutant was severely affected with a C.I. of 2%. A likely explanation is that the *Salmonella* Gcv system does not generate a sufficient C1 flux at the levels of glycine available during infection. In support of this, Stauffer and co-workers [42] found that the Gcv activity in *S.* Typhimurium is only 20% of the level found in *E. coli*, but further studies are needed to clarify whether reduced C1-flux is causing attenuation of the *glyA*-mutant. In conclusion, it appears from the virulent *serA* mutant that serine is available during infection, and in amounts not limiting the C1 flux. However the attenuated *glyA* mutant indicates that glycine is not present in sufficient amounts during infection, for the inefficient Gcv system to fuel the C1-flux. This conclusion requires that *gcv* is indeed functionally active, since alternatively *Salmonella* relies on GlyA for C1-fueling. Studies of gene expression show that the *gvc-*operon is up-regulated in *S.* Typhimurium after 8 hours in cultured macrophages, which suggest that it may be active [7]. Irrespective of the explanation, the observation makes GlyA a promising single target for drug discovery.

Threonine can be synthesized in two ways aside from the threonine biosynthesis pathways. Both of these auxiliary reactions use glycine as precursor. Threonine may be formed in a single step from glycine and acetaldehyde catalyzed by the *ltaA* gene product. Alternatively it may be formed in two steps by the *kbl* and *thr* gene products. In the biosynthetic pathway, threonine is formed from homoserine through the action of ThrB and ThrC. Threonine subsequently functions as a precursor for the synthesis of isoleucine by the action of the *ilvA* – *ilvD* gene products. In a classical genetic analysis, Glanville & Demerec [43] found that a *thrC* mutant in strain LT2 (at that time called a *thrE* mutant) requires either threonine or isoleucine for growth. The fact that isoleucine can support growth of the *thrC* mutant without being converted to threonine is an indication that the threonine flux from glycine is enough to supply threonine-charged tRNAs for protein synthesis. Since the flux of threonine from glycine was not sufficient to support growth of the *thrC* mutant in the absence of Isoleucine, we expected that either the LtaA reaction or the Kbl/Thr path was nonfunctional. We considered the *thrC/kbl* pair suggested by the model analysis as a cut set. In our strain we found that a *thrC* mutant grew somewhat slower than the wild type, while a *kbl* strain had no growth related phenotypes. The *thrC/kbl* double mutant however was unable to grow in minimal medium without threonine supplements, showing that the mutant pair is truly auxotrophic. Interestingly, however, we did not observe any clear infection defect as shown by the C.I. values of either single or double mutants. This confirms that the host must supplies sufficient threonine during all phases of the infection, as recently reported [36].

Approximately 28 reaction pairs predicted by the model analysis are involved in the production of putrescine and/or spermidine (Table S2). Putrescine and spermidine are small polycationic amines of the group polyamines. Putrescine is synthesized via agmatine from arginine by the *speA* and *speB* gene products or directly from ornithine by either of the *speC* or *speF* gene products. Putrescine can subsequently be converted into spermidine by the co-action of the *speE* and *speD* gene products (Figure S4). From the 28 reaction pairs suggested by the model, we analyzed the the effect of combined removal of AGMATIN-RXN and ORNDECOBON-RXN by creating a *speB* mutant, a *speC/speF* double mutant, and a *speB/speC/speF* triple mutant. The triple mutant was not impaired for growth in either rich (LB) or the two M9 media (Table 1) showing that the reactions do not constitute a cut set combination even in the absence of putrescine. However, while a *speB* mutant and a *speC/speF* mutant had close to wild type C.I.’s, the triple mutant had a severe infection defect pointing to the importance of putrescine availability for virulence. We have previously shown that a mutant lacking *speB*, *speC*, *speF* and *speE* is attenuated for virulence signifying that polyamines play a role in virulence of *S.* Typhimurium [29]. However, the quadruple mutant investigated in Jelsbak *et al.* [29] had a reduced growth rate in M9 suggesting that growth rate could be a contributing factor to the virulence attenuation. In the present study, we show that the *speB/speC/speF* triple mutant, which had no growth defect, was still attenuated in mice, thus substantiating the essential roles for polyamines in virulence of *S*. Typhimurium. It has recently been reported that polyamines also contribute to virulence of *Shigella* spp and *Legionella pneumophila*, [44], [45]. Drugs targeting SpeB, SpeC, and SpeF are hence promising candidates for new broad-spectrum antimicrobials.

Finally, *trpA* and *trpB* were predicted to form a cut set (Table 1 and Table S2). They form a bienzyme-complex, which is capable of performing three reactions: 1) the cleavage of indole-3-glycerol phosphate to indole and glyceraldehyde-3-phosphate, 2) the formation of L-tryptophan from L-serine and indole, and 3) the formation of tryptophan from L-serine and indole-2-glycerol phosphate [33]. Activity in *E. coli* has been shown to be significantly higher when these two enzymes work in conjunction rather than as individual enzymes [46]. This observation was confirmed for *S.* Typhimurium in the current investigation, since both the single and the double mutants were growth attenuated in M9+ medium (Figure S1), suggesting that activity of both enzymes are needed for full growth in the medium used as input to the model. Growth was restored to wild type levels when the *trpA* mutant was complemented with *trpA* wild-type allele *in trans*, while *trpB* still showed reduced growth-rate (but reached wild type density) when complemented by the wild type allele *in trans*. As expected, complementation of the double mutant with single genes was unsuccessful. Single gene knock-outs were without impact on fitness *in vivo,* while the double mutant could not compete with the wild type strains. The most obvious explanation for this observation is that tryptophan is not provided by the host during *S.* Typhimurium infection. However, the observation contradicts a previous observation by Steeb *et al.* [36] who considered that tryptophan is not essential for growth of *S.* Typhimurium during infection. In support of our conclusion, Henry *et al.* [47], in a study of genes important for cell division of *S.* Typhimurium in cell cultures, observed that lack of TrpC, which performs the step above the one by TrpB/TrpA in the biosynthesis of trypophane, caused formation of filamentous cells, and thus this enzyme appeared essential for *in vivo* growth. However, further studies are indicated to clarify this matter.

### Concluding remarks

In the present study we have used genome scale metabolic modeling to identify pairs of reactions in *S.* Typhimurium required for intra host survival. Our results revealed that asparagine, tryptophan and most likely glycine are not present in sufficient amounts to support growth while threonine and serine are. These data add to the existing knowledge of available nutrients in the intra-host environment [9]–[12], [36]. In the recent paper by Steeb *et al.* [36], a comprehensive analysis including mutagenesis of intra host expressed enzymes was used to identify available metabolites in the host. Compared to their study, we confirm the availability of threonine, however, in contrast, we found that most likely glycine and tryptophane are only present in limiting amounts, as the *glyA* mutant and the *trpA*;*trpB* double mutant had reduced competitive fitness compared to the wild type. The conclusion regarding the *glyA* mutant should be further substantiated by studies comparing GlyA and GCV performance. Furthermore, we additionally found that asparagine and putrescine are only present in limiting amounts during infection. This is particularly interesting since the SpeB, SpeC, and SpeF enzymes, facilitating putrescine biosynthesis, were not detected in *Salmonella* bacteria isolated from spleens [36]. We have previously reported the essential role of polyamines in virulence of *S.* Typhimurium [29], however, the present results show that polyamines are not required for growth *in vitro*, indicating that they are more specifically related to virulence than to central metabolism. The putrescine biosynthetic genes constitute a novel target for anti-infective therapy with potential of broad effect against several intracellular pathogens.

## Supporting Information

Figure S1 **Growth phenotypes of five selected cut sets in M9 media with supplements (see text).** The media corresponds to the one used as input in the cut set analysis using the *S.* Typhimurium genome scale model.
(PDF)

Figure S2 **The transformation of L-asparatate to L-asparagine catalyzed by the reactions of AsnA and AsnB.** AsnA uses ammonia as a reactant where AsnB predominantly uses glutamine.
(PDF)

Figure S3 **The biosynthesis of serine.** Serine can be produced from glycine through the action of GlyA and also from 3-phospo-glycecerate (3PG) by the action of SerA/B/C.
(PDF)

Figure S4 **The polyamine biosynthesis pathways in** ***Salmonella*** **.** Putrescine can be formed from arginine through the reactions catalyzed by SpeA and SpeB and from ornithine through the reaction catalyzed by SpeC/SpeF. Spermidine is formed from putrescine through the reactions of SpeE and SpeD.
(PDF)

Table S1 **Primers used for construction of mutants and for cloning purposes.**
(PDF)

Table S2 **A list of 102 reaction pairs identified as unfeasible for production of essential biomass in the damage analysis.**
(PDF)
