# Supplementary material for: Identification of Metabolic Pathways Essential for Fitness of Salmonella Typhimurium In Vivo
Source: PLoS One. 2014 Jul 3;9(7):e101869. doi: 10.1371/journal.pone.0101869 (PMC4081726; doi:10.1371/journal.pone.0101869)

# Growth of *ΔasnA*, *ΔasnB* and *ΔasnA;ΔasnB* in M9+aa

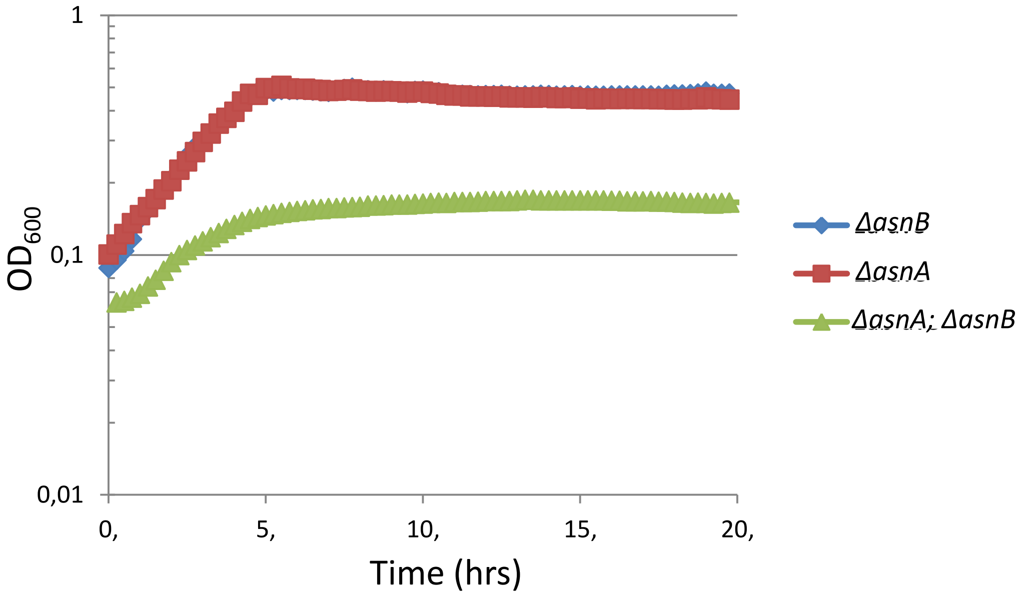

# Growth of *ΔserA*, *ΔglyA* and *ΔserA;ΔglyA* in M9+aa

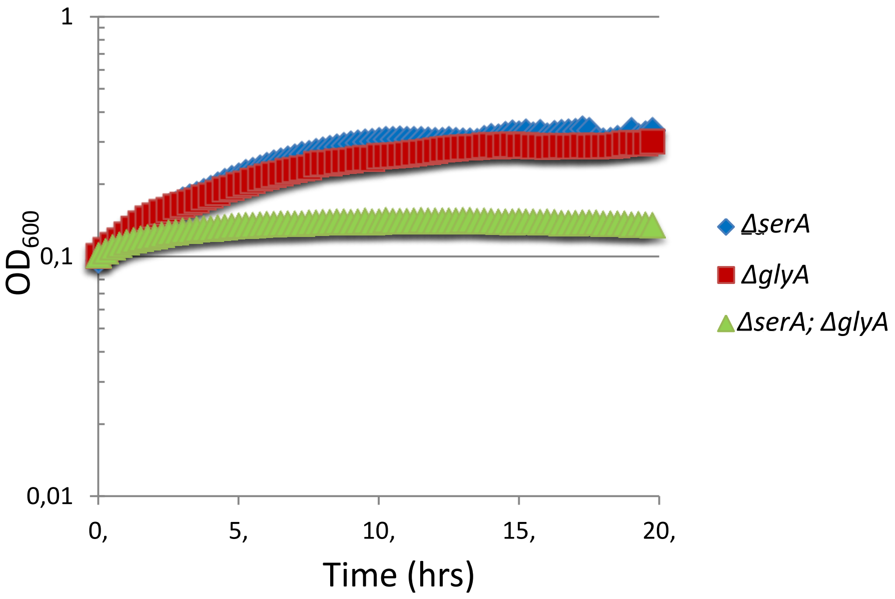

# Growth of *ΔspeB*, *ΔspeC; ΔspeF* and *ΔspeB;ΔspeC; ΔspeF* in M9+aa

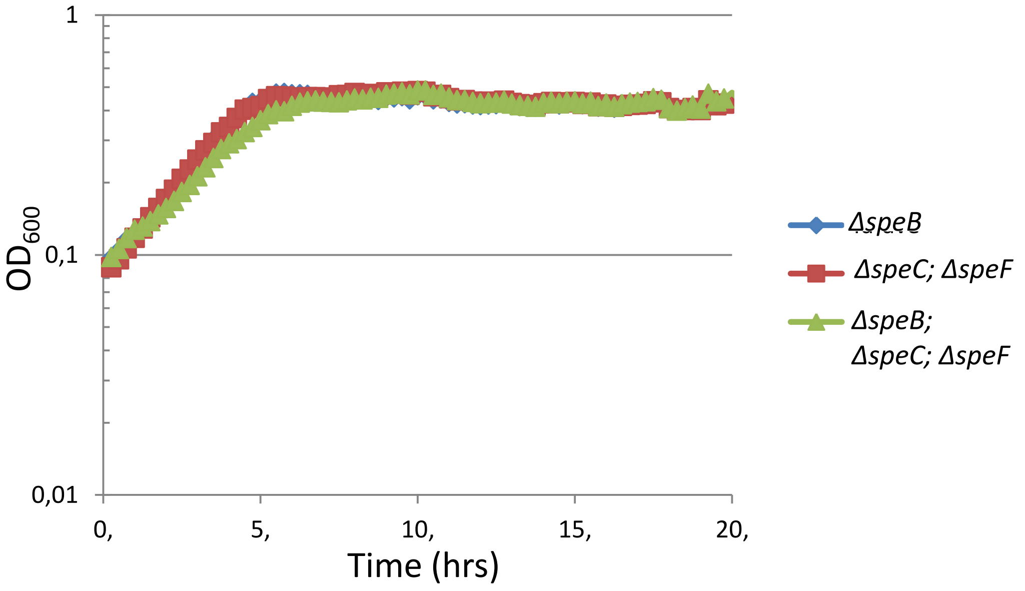

# Growth of *ΔthrC*, *Δkbl* and *ΔthrC;Δkbl* in M9+aa

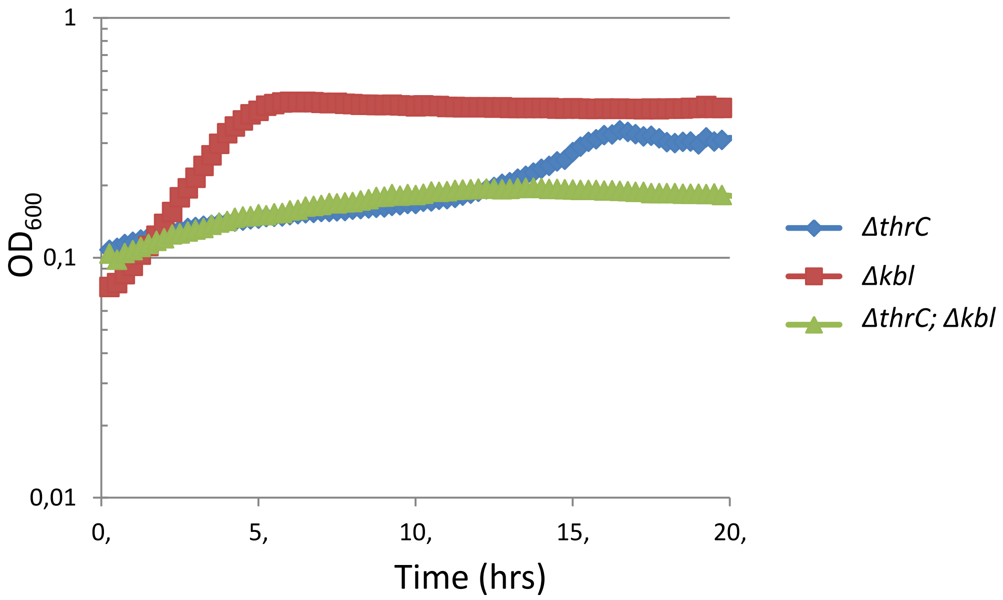

# Growth of *ΔtrpA*, *ΔtrpB, ΔtrpA;ΔtrpB* and complemented strains in M9+aa

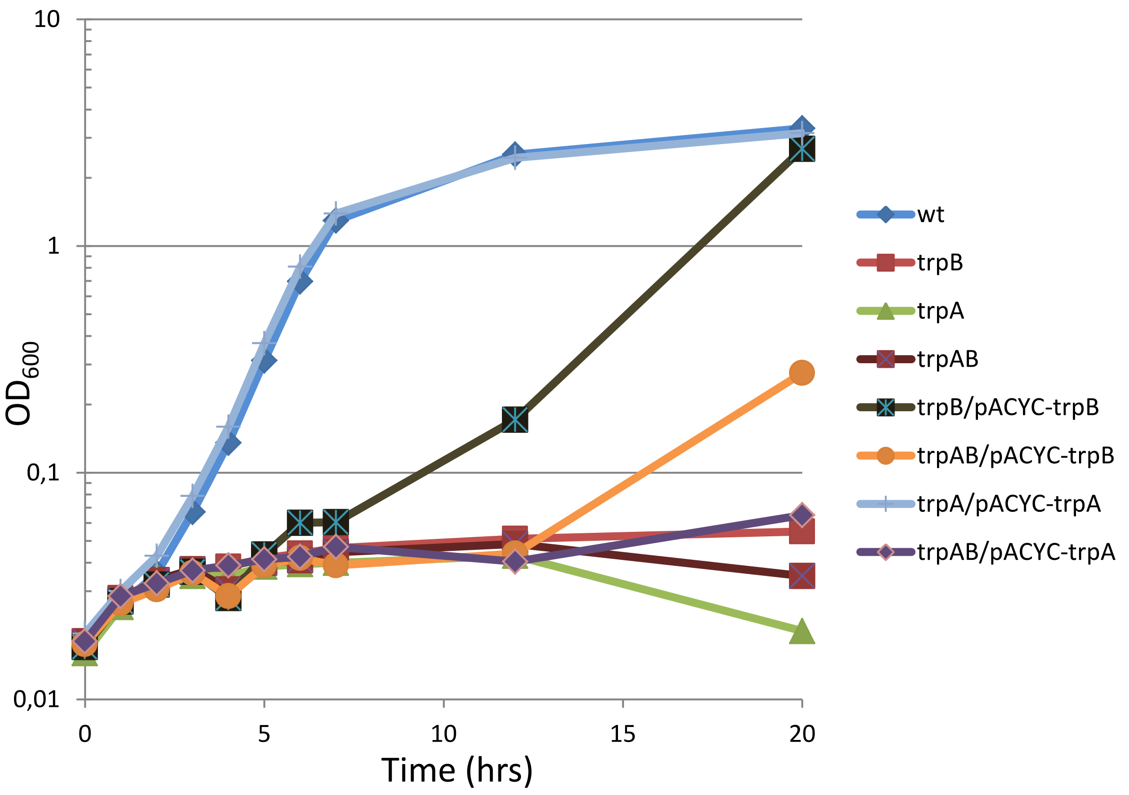


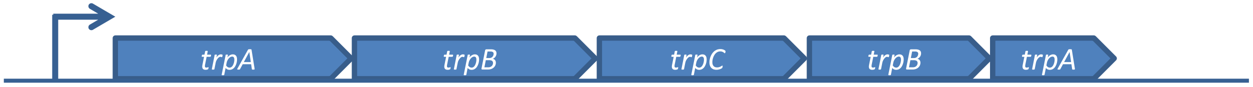

Supplement: Figure S1 — Growth phenotypes of five selected cut sets in M9 media with supplements (see text). The media corresponds to the one used as input in the cut set analysis using the S. Typhimurium genome scale model. (PDF) [file pone.0101869.s001.pdf]
