# Supplementary material for: Identification of Metabolic Pathways Essential for Fitness of Salmonella Typhimurium In Vivo
Source: PLoS One. 2014 Jul 3;9(7):e101869. doi: 10.1371/journal.pone.0101869 (PMC4081726; doi:10.1371/journal.pone.0101869)

Supplementary Figure S2. The transformation of L-asparatate to L-asparagine catalyzed by the reactions of AsnA and AsnB. AsnA uses ammonia as a reactant where AsnB predominantly uses glutamine.

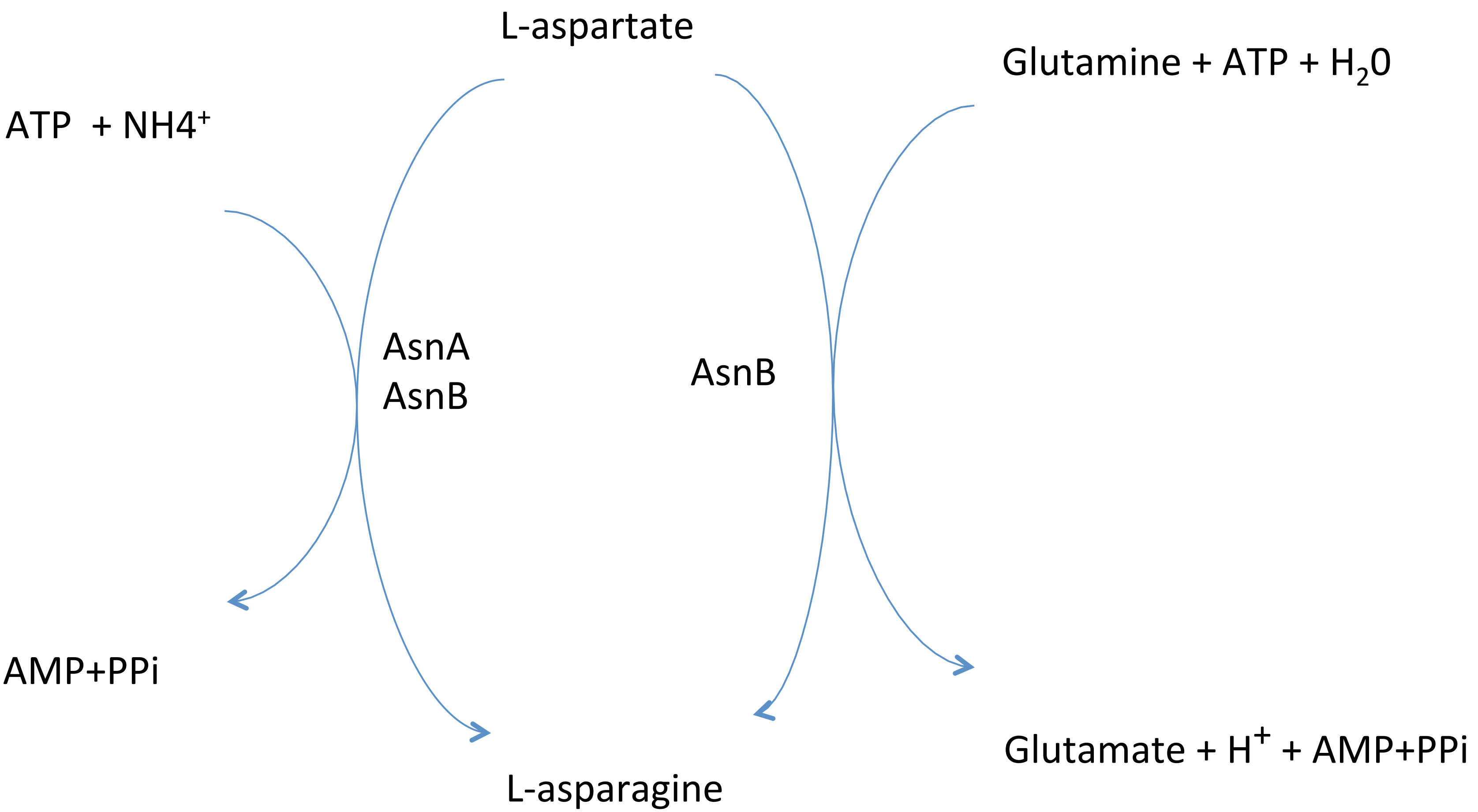

Supplement: Figure S2 — The transformation of L-asparatate to L-asparagine catalyzed by the reactions of AsnA and AsnB. AsnA uses ammonia as a reactant where AsnB predominantly uses glutamine. (PDF) [file pone.0101869.s002.pdf]
