# Supplementary material for: Identification of Metabolic Pathways Essential for Fitness of Salmonella Typhimurium In Vivo
Source: PLoS One. 2014 Jul 3;9(7):e101869. doi: 10.1371/journal.pone.0101869 (PMC4081726; doi:10.1371/journal.pone.0101869)
Supplement: Figure S3 — The biosynthesis of serine. Serine can be produced from glycine through the action of GlyA and also from 3-phospo-glycecerate (3PG) by the action of SerA/B/C. (PDF) [file pone.0101869.s003.pdf]

Supplementary Figure 3. The biosynthesis of serine. Serine can be produced from glycine through the action of GlyA and also from 3-phospo-glycecerate (3PG) by the action of SerA/B/C.

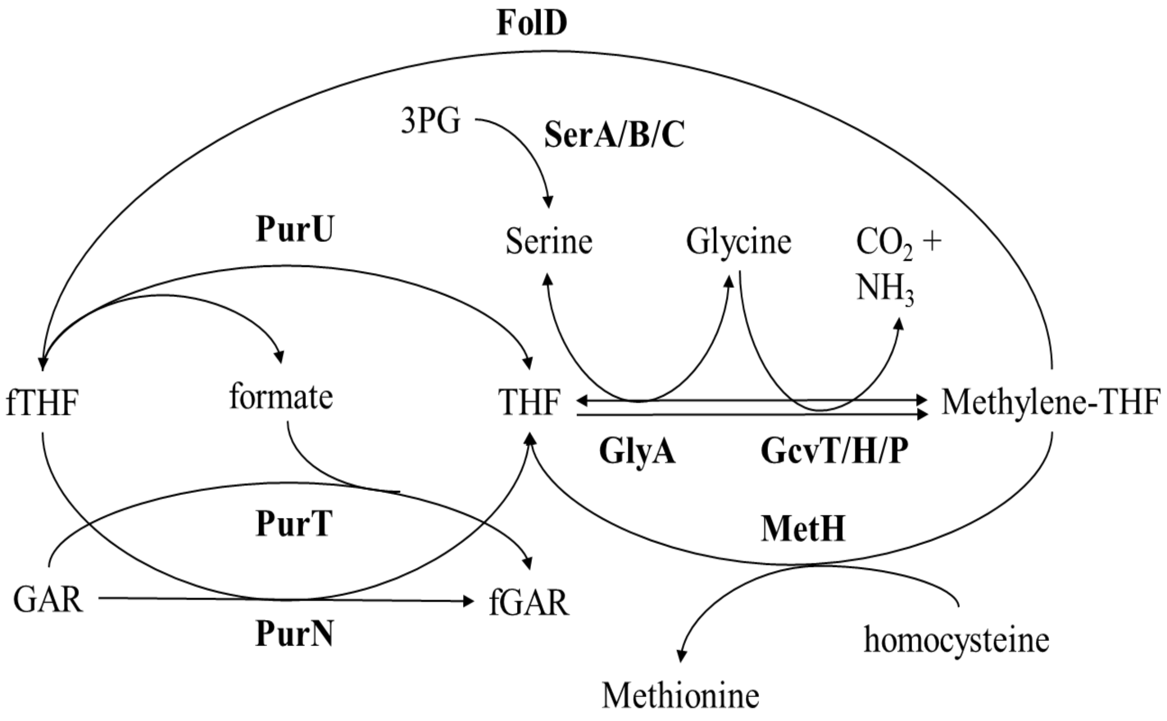

[illegible]
