# Supplementary material for: Identification of Metabolic Pathways Essential for Fitness of Salmonella Typhimurium In Vivo
Source: PLoS One. 2014 Jul 3;9(7):e101869. doi: 10.1371/journal.pone.0101869 (PMC4081726; doi:10.1371/journal.pone.0101869)

Supplementary Figure S4. The polyamine biosynthesis pathways in *Salmonella*. Putrescine can be formed from arginine through the reactions catalyzed by SpeA and SpeB and from ornithine through the reaction catalyzed by SpeC/SpeF. Spermidine is formed from putrescine through the reactions of SpeE and SpeD.

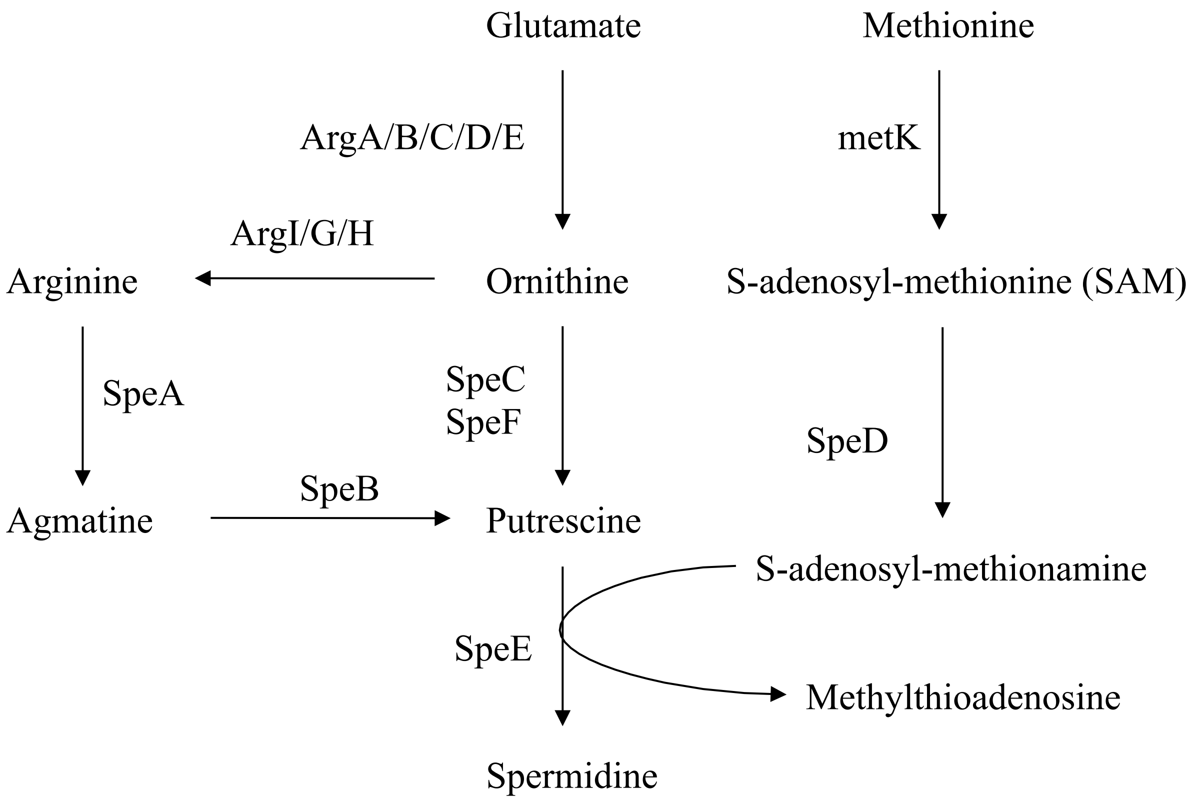

Supplement: Figure S4 — The polyamine biosynthesis pathways in Salmonella . Putrescine can be formed from arginine through the reactions catalyzed by SpeA and SpeB and from ornithine through the reaction catalyzed by SpeC/SpeF. Spermidine is formed from putrescine through the reactions of SpeE and SpeD. (PDF) [file pone.0101869.s004.pdf]
