# Supplementary material for: Identification of Metabolic Pathways Essential for Fitness of Salmonella Typhimurium In Vivo
Source: PLoS One. 2014 Jul 3;9(7):e101869. doi: 10.1371/journal.pone.0101869 (PMC4081726; doi:10.1371/journal.pone.0101869)
Supplement: Table S1 — Primers used for construction of mutants and for cloning purposes. (PDF) [file pone.0101869.s005.pdf]

Table S1, Primers used to construct mutants and complementation plasmids

Primer name	Sequence	Application
STM1780fwd	GTACGCGGTGGTGATATTTTCATCATCCAGTCCACTTGgtgtaggctggagctgcttc	Mutant construction
STM1780rev	CGCAGGTTGTTTGCCGCATTGCCTGAGAAGATCGGGTGcatatgaatatcctccttag	Mutant construction
STM3877fwd	TGTTGCTTAATCATAGGCAACAGGACGCAGGAGTAAAAAACATATGAATATCCTCCTTAG	Mutant construction
STM3877rev	ATGCGAAGCCGCCTGCTGAGACGCTGGCGGCGCTAACGGCTGTGTAGGCTGGAGCTGCTT	Mutant construction
STM0680fwd	AATACAAAAAGTACGACGCAACAGATTAACGGAGAAGGCTCATATGAATATCCTCCTTAG	Mutant construction
STM0680rev	TAAAAAAAGAGCCCCTAAGGGCTCTTTCGTCATCGTTAATTGTGTAGGCTGGAGCTGCTT	Mutant construction
STM3062fwd	GGTGATTGGCGAGCTATTGCTGCTGCTGCGCGGCGTGCCGgtgtaggctggagctgcttc	Mutant construction
STM3062rev	TTGCAGATACTGTGCGGCAATGTTAACGCCCTGCTCGGCcatatgaatatcctccttag	Mutant construction
STM3062con	GGAAAGCCTTCGTGCGGCAGGTTACACC	Mutant verifcation
STM2555fwd	TGGGTATGAACCTGGCGCAGGGCGGCCACCTGACTCACgtgtaggctggagctgcttc	Mutant construction
STM2555rev	ACCGCCGGAGAACCAATACGGATACCGGAGGTCACGAACcatatgaatatcctccttag	Mutant construction
STM2555con	GATCGCCTCCGAAAACTACACCAGCCCG	Mutant verifcation
STM0004fwd	CCGCAGCAAATTCTGGAAGAGCGCGTCCGCGCGGCGTTgtgtaggctggagctgcttc	Mutant construction
STM0004rev	TCGCTTTCAGCTCGCGCATCGTCTGCTGTGTCGTAGTAcatatgaatatcctccttag	Mutant construction
STM0004con	AATGAGCAGGTCAGCTTTGCGCAGGCCG	Mutant verifcation
STM3709fwd	GTTTCGGTATGGCGTCCGTGCGTTTTATCTGCGGCACCgtgtaggctggagctgcttc	Mutant construction
STM3709rev	ATCCGCGCCTGCCAGCGTAAATCCCGCGGCAGACATCTcatatgaatatcctccttag	Mutant construction
STM3709con	TACGTCTGCGCAGCAGGCGGATATCACC	Mutant verifcation
STM1726fwd	TACCACGTTGTATTTAAAGCGCGAAGATTTACTGCACGgtgtaggctggagctgcttc	Mutant construction
STM1726rev	GACCACCAGCAGTTGCTCTTTTTCCGGCTGCTCGCGCATCcatatgaatatcctccttag	Mutant construction
STM1726con	GGTGAATTCGGCGGCATGTATGTGCCGC	Mutant verifcation
STM1727fwd	GGGGGTTCCCTTCTCCGATCCGCTGGCCGATGGCCCTAgtgtaggctggagctgcttc	Mutant construction
STM1727rev	CGAGGAGATACCGAAGCCCTGTAACGCAGGCGCGGCATcatatgaatatcctccttag	Mutant construction
STM1727con	AACGATCGCCGGGAAGGCGCTTTTGTCC	Mutant verifcation
Kanrev	CCGCTTCAGTGACAACGTCGAGCACAGC	Mutant verifcation
Camfwdny	TACGCAAGGCGACAAGGTGCTGATGCCG	Mutant verifcation
STM3877-fwd	CCAActgcagcggccgctactagtaTTATAAAATAGCAGGAATGCTTTCGCG	Complementation
STM3877-rev	GGTTgaattcgcggccgcttctagagCGGTCCAGATTGTCGATCTGA	Complementation
STM2555-fwd-XhoI	ctctcgagATTGTTAGCTGAGTCAGGAGATGCGG	Complementation
STM2555-rev-BamHI	ctggatccGCATAAGTAATGCCCGACAGACGCAG	Complementation
STM1726compfwd	ctctcgagCCGGGCATCAAAGATGCTCGTCTTCTGG	Complementation
STM1726comprev	ctggatccTCAGTGACTGTTCAATGCCAGGGTCGCC	Complementation
STM1727compfwdny	ctctcgagCGTTGGCGCACGCTCTGAAAATGATGCG	Complementation
STM1727comprevny	ctggatccTGAATCGTGATTCTGGTTCGGCGCCAGC	Complementation
