# Supplementary material for: Identification of Metabolic Pathways Essential for Fitness of Salmonella Typhimurium In Vivo
Source: PLoS One. 2014 Jul 3;9(7):e101869. doi: 10.1371/journal.pone.0101869 (PMC4081726; doi:10.1371/journal.pone.0101869)
Supplement: Table S2 — A list of 102 reaction pairs identified as unfeasible for production of essential biomass in the damage analysis. (PDF) [file pone.0101869.s006.pdf]

Supplementary Table S2. List of 102 reaction pairs identified by damage analysis

Predition number	Redundant reactions	Genes	Metabolites predicted to be affected
1	RIB5PISOM-RXN<br><br>TRANSALDOL-RXN	STM3063 (*rpiA)*, STM1933 (*yedJ*)<br>STM4109 (*talC*), STM2473 (*talA*), STM0007 (*talB*)	nadh, Mthf, Succoa, nad, Coa, AcCoA, His, nadp, UDP-Glucose, dATP, fad, Spermidine, nadph, gtp, amp, utp, ctp, atp, Trp, dCTP, lps, dGTP
2	PGLYCDEHYDROG-RXN<br><br>GLYOHMETRANS-RXN	STM3062 (*serA*)<br>STM2555 (*glyA*)	Phosphatidylethanolamine, Ser, Trp, Phosphatidylserin
3	GART-RXN<br><br>GARTRANSFORMYL2-RXN	STM2500 (*purN*)<br>STM1883 (*purT)*	nadh, Mthf, Succoa, nad, Coa, AcCoA, nadp, dATP, fad, nadph, gtp, amp, atp, dGTP
4	DURIDKI-RXN<br><br>DUTP-PYROP-RXN	STM1750 (*tdk*)<br>STM3731 (*dut*)	dTTP
5	ACETYLGLUTKIN-RXN<br>3.5.2.14-RXN	STM4122*(argB*)	Putrescine, Spermidine
6	RIB5PISOM-RXN<br><br>RIBULP3EPIM-RXN	STM3063 (*rpiA*), STM1933 (hypothetical protein)<br>STM1617 (epimerase), STM4080 (epimerase), STM3483 (*rpe*)	nadh, Mthf, Succoa, nad, Coa, AcCoA, His, nadp, UDP-Glucose, dATP, fad, Spermidine, nadph, gtp, Phe, amp, utp, Tyr, ctp, atp, Trp, dCTP, lps, dGTP
7	ACETYLGLUTKIN-RXN<br>CREATININE-DEAMINASE-RXN	STM4122 (*argB)*<br>STM3334 (cytocine deaminase)	Putrescine, Spermidine
8	INOPHOSPHOR-RXN<br><br>PRPPSYN-RXN	STM1780 (*prsA*)	nadh, Mthf, Succoa, nad, Coa, AcCoA, His, nadp, UDP-Glucose, dATP, fad, Spermidine, nadph, gtp, amp, utp, ctp, atp, Trp, dCTP, dGTP
9	RXN-7968-(NAD)<br><br>SHIKIMATE-5-DEHYDROGENASE-RXN	STM1359 (*aroE*)<br>STM1359 (*aroE*), STM3401 (*aroE*), STM3859 (*aroE*)	Mthf, Phe, Tyr, Trp
10	ACETYLORNTRANSAM-RXN<br><br>CREATINASE-RXN	STM3468 (*argD*)	Putrescine, Spermidine

11	AGMATIN-RXN<br>ORNDECARBOX-RXN	STM3078 (*speB*)<br>STM0701 (*speF*), STM3114 (*speC*)	Putrescine, Spermidine
12	RIB5PISOM-RXN<br><br>2TRANSKETO-RXN	STM3063 (*rpiA*), STM1933 (hypothetical protein)<br>STM2340 (transketolase), STM2341 (transketolase), STM2474 (*tktB*), STM3076 (*tktA*)	nadh, Mthf, Succoa, nad, Coa, AcCoA, His, nadp, UDP-Glucose, dATP, fad, Spermidine, nadph, gtp, Phe, amp, utp, Tyr, ctp, atp, Trp, dCTP, lps, dGTP
13	QUINOLINATE-SYNTHE-MULTI-RXN<br>QUINOLINATE-SYNTHA-RXN	STM0756 (*nadA*)<br>STM0756 (*nadA)*	nadh, nad, nadp, nadph
14	THRESYN-RXN<br><br>AKBLIG-RXN	STM0004 (*thrC*)<br>STM3709 (*kbl*)	Ile, Thr
15	ADENYL-KIN-RXN<br><br>INOSINEKIN-RXN	STM0488 (*adk*)<br>STM0491 (*gsk*)	nadh, Mthf, Succoa, nad, Coa, AcCoA, His, Asn, nadp, UDP-Glucose, dATP, fad, Cys, Spermidine, nadph, gtp, amp, utp, ctp, atp, Trp, dCTP, Met, dGTP
16	RXN0-5114<br><br>GLYOHMETRANS-RXN	STM2197 (phosphoserine-phosfortase), STM4578 (*serB*)<br>STM2555 (*glyA*)	Phosphatidylethanolamine, Ser, Trp, Phosphatidylserin
17	AKBLIG-RXN<br><br>HOMOSERKIN-RXN	STM3709 (*kbl*)<br>STM0003 (*thrB*)	Ile, Thr
18	1TRANSKETO-RXN<br><br><br><br>RIBULP3EPIM-RXN	STM2340 (transketolase), STM2341 (transketlase), STM2474 (*tktB*), STM3076 (*tktA*)<br>STM1617 (epimerase), STM4080 (epimerase), STM3483 (*rpe)*	Mthf, Phe, Tyr, Trp, lps
19	RXN-10639<br><br><br>PHOSPHOGLUCMUT-RXN	 <br>STM2820 (*ygaBk*), STM0698 (*pgm*)	Star, UDP-Glucose, lps
20	1TRANSKETO-RXN	STM2340 (transketolase), STM2341 (transketlase),	Mthf, Phe, Tyr, Trp, lps

	2TRANSKETO-RXN	STM2474 (*tktB*), STM3076 (*tktA*)<br>STM2340 (transketolase), STM2341 (transketlase), STM2474 (*tktB*), STM3076 (*tktA*)	
21	LCYSDESULF-RXN<br><br>ACSERLY-RXN	STM2430 (transketolase), STM2440 (*cysM*)	Succoa, Coa, AcCoA, Cys, Spermidine, Met
22	ARGDECARBOX-RXN<br><br>ORNDECARBOX-RXN	STM4296 (*adi*), STM3086 (*speA*)<br>STM0701, STM3114	Putrescine, Spermidine
23	F16BDEPHOS-RXN<br><br>Glc_tx	STM4415 (*fbp*)	nadh, Mthf, Succoa, nad, Coa, AcCoA, Star, His, nadp, Peptidoglycan, UDP-Glucose, dATP, fad, Spermidine, nadph, gtp, Phe, amp, utp, Tyr, ctp, atp, Trp, dCTP, lps, dGTP
24	ACETYLGLUTKIN-RXN<br>CREATINASE-RXN	STM4122 (*argB*)	Putrescine, Spermidine
25	RIB5PISOM-RXN<br><br>RXN-3341	STM3063 (*rpiA*), STM1933 (hypothetical protein)<br>STM2081 (*gnd*), STM2083 (*rfbK*), STM2084 (*rfbM*), STM2091 (*rfbG*), STM2092 (*rfbF*)	nadh, Mthf, Succoa, nad, Coa, AcCoA, His, nadp, UDP-Glucose, dATP, fad, Spermidine, nadph, gtp, Phe, amp, utp, Tyr, ctp, atp, Trp, dCTP, lps, dGTP
26	RIB5PISOM-RXN<br><br>KDPGALDOL-RXN	STM3063 (*rpiA*), STM1933 (hypothetical protein)<br>STM1884 (*eda*)	nadh, Mthf, Succoa, nad, Coa, AcCoA, His, nadp, UDP-Glucose, dATP, fad, Spermidine, nadph, gtp, Phe, amp, utp, Tyr, ctp, atp, Trp, dCTP, lps, dGTP
27	ORNDECARBOX-RXN<br>CREATININASE-RXN	STM0701 (*speF*), STM3114 (*speC*)	Putrescine, Spermidine
28	INOPHOSPHOR-RXNINORGPYROPHOSPHAT-RXN	STM4414 (is not found in KEGG)	nadh, Mthf, Succoa, nad, Phosphatidylethanolamine, Coa, AcCoA, Star, His, Asn, nadp, Peptidoglycan, UDP-Glucose, Phosphatidylglycerol, dATP, fad, Cys, Spermidine, nadph, gtp, amp, utp, ctp, atp, Trp, clpn, Phosphatidylserin, dCTP,

			Met, lps, dGTP
29	ADENPRIBOSYLTRAN-RXN<br><br>PRPPSYN-RXN	STM0483 (*apt*)<br>STM1780 (*prsA*)	nadh, Mthf, Succoa, nad, Coa, AcCoA, His, nadp, UDP-Glucose, dATP, fad, nadph, gtp, amp, utp, ctp, atp, Trp, dCTP, dGTP
30	RIBULP3EPIM-RXN<br><br><br>GLU6PDEHYDROG-RXN	STM1617 (epimerase), STM4080, (epimerase)<br>STM3483 (*rpe|)*<br>STM1886 (*zwf*)	nadh, Mthf, Succoa, nad, Coa, AcCoA, His, nadp, UDP-Glucose, dATP, fad, Spermidine, nadph, gtp, Phe, amp, utp, Tyr, ctp, atp, Trp, dCTP, lps, dGTP
31	GLUCOSAMINE-6-P-DEAMIN-RXN<br>L-GLN-FRUCT-6-P-AMINOTRANS-RXN	STM0684 (*nagB*)<br><br>STM4539 and STM 4540) (glucosamine-fructose-6-phosphate aminotransferase), STM3861 (*glmS*)	Peptidoglycan, lps
32	ARGSUCCINSYN-RXN<br><br>arginine_tx	STM3290 (*argG*)	Arg
33	ORNCARBAMTRANSFER-RXN<br><br><br><br>arginine_tx	STM4465 (ornithine carbamoyltransferase), STM4469 (*argL*)	Arg
34	N-ACETYLGLUTPREDUCT-RXN<br>N-CARBAMOYLSARCOSINE-AMIDASE-RXN	STM4121 (*argC*)	Putrescine, Spermidine
35	F16ALDOLASE-RXN<br><br>Glc_tx	STM3780 (*gatY*), STM4022 (*yihT*), STM4078 (*yneB*), STM3068 (*fba*), STM2141 (*fbaB*)	nadh, Mthf, Succoa, nad, Coa, AcCoA, Star, His, nadp, Peptidoglycan, UDP-Glucose, dATP, fad, Spermidine, nadph, gtp, Phe, amp, utp, Tyr, ctp, atp, Trp, dCTP, lps, dGTP
36	RXN0-2381<br><br>TRYPSYN-RXN	STM1727 (*trpA*)<br>STM1726 (*trpA*), STM1727 (*trpB*)	Trp

37	1TRANSKETO-RXN<br><br>TRANSALDOL-RXN	STM2340 (transketolase), STM2341 (transketlase), STM2474 (*tktB*), STM3076 (*tktA*)<br>STM4109 (*talC*), STM2473 (*talA*), STM0007 (*talB*)	lps
38	SUCCCOASYN-RXN<br><br>2OXOGLUTARATEDEH-RXN	STM0738 (*sucC*), STM0739 (*sucD*)	Succoa, Peptidoglycan, Lys
39	TRIOSEPISOMERIZATION-RXN<br>Glc_tx	STM4081 (*tpiA*)	nadh, Mthf, Succoa, nad, Phosphatidylethanolamine, Coa, AcCoA, Star, His, nadp, Peptidoglycan, UDP-Glucose, Phosphatidylglycerol, dATP, fad, Spermidine, nadph, gtp, Phe, amp, DGR, utp, Phosphatidate, Tyr, ctp, atp, Trp, clpn, Phosphatidylserin, dCTP, lps, dGTP
40	O2_tx<br><br>SUCCINATE-DEHYDROGENASE-UBIQUINONE-RXN	STM0732 (*sdhC*), STM0733 (*sdhD*), STM0734 (*sdhA*), STM0735 (*sdhB*)	UDP-Glucose, utp, dTTP, ctp, dCTP
41	ADENYL-KIN-RXN<br><br>PRPPSYN-RXN	STM0488 (*adk*)<br>STM1780 (*prsA*)	nadh, Mthf, Succoa, nad, Coa, AcCoA, His, Asn, nadp, dATP, fad, Cys, Spermidine, nadph, atp, Met
42	ARGSUCCINLYA-RXN<br><br>arginine_tx	STM4123 (*argH*)	Arg
43	ISPH2-RXN-(NADP)<br><br>ISPH2-RXN-(NAD)	STM0049 (*ispH*)<br>STM0049 (*ispH*)	Peptidoglycan
44	ADENPRIBOSYLTRAN-RXN<br>ADENYL-KIN-RXN	STM0483 (*apt*)<br>STM0488 (*adk*)	nadh, Mthf, Succoa, nad, Coa, AcCoA, His, Asn, nadp, UDP-Glucose, dATP, fad, Cys, Spermidine, nadph, gtp, amp, utp, ctp, atp, Trp, dCTP, Met, dGTP
45	CREATINASE-RXN<br><br>ORNDECARBOX-RXN	STM0701 (*speF*), STM3114 (*speC*)	Putrescine, Spermidine

46	F16ALDOLASE-RXN<br><br>TRIOSEPISOMERIZATION-RXN	STM3780 (*gatY*), STM4022 (*yihT*), STM4078 (*yneB*), STM3068 (*fba*), STM2141 (*fbaB*)<br>STM4081 (*tpiA*)	nadh, nad, Phosphatidylethanolamine, nadp, Phosphatidylglycerol, nadph, DGR, Phosphatidate, clpn, Phosphatidylserin
47	PHOSPHOGLUCMUT-RXN<br><br>BETA-PHOSPHOGLUCOMUTASE-RXN	STM2820 (*ygaB*), STM0698 (*pgm*)	Star, UDP-Glucose, lps
48	PHOSGLYPHOS-RXN<br><br>Glc_tx	STM3069 (*pgk*)	nadh, Mthf, Succoa, nad, Phosphatidylethanolamine, Coa, AcCoA, Star, His, nadp, Peptidoglycan, UDP-Glucose, Phosphatidylglycerol, dATP, fad, Spermidine, nadph, gtp, Phe, amp, DGR, utp, dTTP, Phosphatidate, Tyr, ctp, atp, Trp, clpn, Phosphatidylserin, dCTP, lps, dGTP
49	INORGPYROPHOSPHAT-RXN<br><br>INOSINEKIN-RXN	STM4414 (*ppa*)<br>STM0491 (*gsk*)	nadh, Mthf, Succoa, nad, Phosphatidylethanolamine, Coa, AcCoA, Star, His, Asn, nadp, Peptidoglycan, UDP-Glucose, Phosphatidylglycerol, dATP, fad, Cys, Spermidine, nadph, gtp, amp, utp, ctp, atp, Trp, clpn, Phosphatidylserin, dCTP, Met, lps, dGTP
50	THRESYN-RXN<br><br>THREODEHYD-RXN	STM0004 (*thrC*)<br>STM3708 (*gatY*)	Ile, Thr
51	ACETYLORNTRANSAM-RXN<br><br>3.5.2.14-RXN	STM3468 (*argD*)	Putrescine, Spermidine
52	GAPOXNPHOSPHN-RXN<br>Glc_tx	STM1290 (*yeaL*)	nadh, Mthf, Succoa, nad, Phosphatidylethanolamine, Coa, AcCoA, Star, His, nadp, Peptidoglycan, UDP-Glucose, Phosphatidylglycerol, dATP, fad, Spermidine, nadph, gtp, Phe, amp, DGR, utp, dTTP,

			Phosphatidate, Tyr, ctp, atp, Trp, clpn, Phosphatidylserin, dCTP, lps, dGTP
53	PGLUCISOM-RXN<br><br>Glc_tx	STM4221 (*pgi*)	Star, UDP-Glucose, lps
54	URACIL-PRIBOSYLTRANS-RXN<br>DUTP-PYROP-RXN	STM2498 (*uup*)<br>STM3731 (*dut*)	dTTP
55	NAD-SYNTH-NH3-RXN<br><br>NAD-SYNTH-GLN-RXN	STM1310 (*nadE*)<br>STM1310 (*nadE*)	nadh, nad, nadp, nadph
56	PPENTOMUT-RXN<br><br>PRPPSYN-RXN	STM4569 (*deoB*)<br>STM1780 (*prsA*)	nadh, Mthf, Succoa, nad, Coa, AcCoA, His, nadp, UDP-Glucose, dATP, fad, Spermidine, nadph, gtp, amp, utp, ctp, atp, Trp, dCTP, dGTP
57	N-ACETYLGLUTPREDUCT-RXN<br>CREATININASE-RXN	STM4121 (*argC*)	Putrescine, Spermidine
57	6PGLUCONOLACT-RXN<br>RIBULP3EPIM-RXN	STM0785 (*ybhE*)<br>STM1617 (epimerase),<br>STM4080 (epimerase),<br>STM3483 (*rpe*)	nadh, Mthf, Succoa, nad, Coa, AcCoA, His, nadp, UDP-Glucose, dATP, fad, Spermidine, nadph, gtp, Phe, amp, utp, Tyr, ctp, atp, Trp, dCTP, lps, dGTP
59	HISTALDEHYD-RXN<br><br>RXN-8001	STM2072 (*hisD*)<br>STM2072 (*hisD*)	His
60	LCYSDESULF-RXN<br>SERINE-O-ACETTRAN-RXN	 STM3699 (*cysE*)	Succoa, Coa, AcCoA, Cys, Spermidine, Met
61	URA-PHOSPH-RXN<br><br>DUTP-PYROP-RXN	STM4568 (*deoA*)<br>STM3731 (*dut*)	dTTP
62	INORGPYROPHOSPHAT-RXN<br><br>PRPPSYN-RXN	STM4414 (*ppa*)<br>STM1780 (*prsA*)	nadh, Mthf, Succoa, nad, Phosphatidylethanolamine, Coa, AcCoA, Star, His, Asn, nadp, Peptidoglycan, UDP-Glucose, Phosphatidylglycerol, fad, Cys, Spermidine, nadph, clpn, Phosphatidylserin, Met, lps

63	O-SUCCHOMOSERLYASE-RXNCYSPH-RXN	STM3624A (cystathionine gamma-synthase), STM4100 (*metB*) STM3624A (cystathionine gamma-synthase)	Spermidine, Met
64	INOPHOSPHOR-RXN<br><br>ADENYL-KIN-RXN	STM0488 (*adk*)	nadh, Mthf, Succoa, nad, Coa, AcCoA, His, Asn, nadp, UDP-Glucose, dATP, fad, Cys, Spermidine, nadph, gtp, amp, utp, ctp, atp, Trp, dCTP, Met, dGTP
65	RIB5PISOM-RXN<br><br>PGLUCONDEHYDRAT-RXN	STM3063 (*rpiA*), STM1933 (hypothetical protein) STM1885 (*edd*)	nadh, Mthf, Succoa, nad, Coa, AcCoA, His, nadp, UDP-Glucose, dATP, fad, Spermidine, nadph, gtp, Phe, amp, utp, Tyr, ctp, atp, Trp, dCTP, lps, dGTP
66	INORGPYROPHOSPHAT-RXN<br><br>PPENTOMUT-RXN	STM4414 (*ppa*)<br>STM4569 (*deoB*)	nadh, Mthf, Succoa, nad, Phosphatidylethanolamine, Coa, AcCoA, Star, His, Asn, nadp, Peptidoglycan, UDP-Glucose, Phosphatidylglycerol, dATP, fad, Cys, Spermidine, nadph, gtp, amp, utp, ctp, atp, Trp, clpn, Phosphatidylserin, dCTP, Met, lps, dGTP
67	N-ACETYLGLUTPREDUCT-RXN<br>CREATININE-DEAMINASE-RXN	STM4121 (*argC*)<br>STM3334 (cytocine deaminase)	Putrescine, Spermidine
68	INOSINEKIN-RXN<br><br>PRPPSYN-RXN	STM0491 (*gsk*)<br>STM1780 (*prsA*)	nadh, Mthf, Succoa, nad, Coa, AcCoA, His, nadp, UDP-Glucose, dATP, fad, Spermidine, nadph, gtp, amp, utp, ctp, atp, Trp, dCTP, dGTP
69	CARBAMATE-KINASE-RXN<br><br>RXN0-5224	STM4466 (carbamate kinase), STM0532 (*argC*) PSLT046 (putative carbonic anhydrase), STM0171 (*yadF*)	UDP-Glucose, utp, dTTP, ctp, dCTP
70	2TRANSKETO-RXN<br><br>TRANSALDOL-RXN	STM2340 (transketolase), STM2341 (transketlase), STM2474 (*tktB*), STM3076 (*tktA*)<br>STM4109 (*talC*), STM2473 (*talA*), STM0007 (*talB*)	Mthf, Phe, Tyr, Trp

71	ORNDECARBOX-RXN<br><br>CREATININE-DEAMINASE-RXN	STM0701 (*speF*), STM3114 (*speC*)<br>STM3334 (cytosine deaminase)	Putrescine, Spermidine
72	ASNSYNB-RXN<br>ASNSYNA-RXN	STM0680 (*asnB*)<br>STM3877 *asnA*)	Asn
73	3.5.2.14-RXN<br><br>ORNDECARBOX-RXN	 STM0701 (*speF*), STM3114 (*speC*)	Putrescine, Spermidine
74	O-SUCCHOMOSERLYASE-RXN<br>HOMOSERKIN-RXN	STM3624A (cystathionine gamma-synthase)<br>STM4100 (*metB*)<br>STM0003 (*thrB*)	Spermidine, Met
75	RIBULP3EPIM-RXN<br><br>TRANSALDOL-RXN	STM1617 (epimerase), STM4080, (epimerase)<br>STM3483 (*rpe|)*<br>STM4109 (*talC*), STM2473 (*talA*), STM0007 (*talB*)	Mthf, Phe, Tyr, Trp
76	N-CARBAMOYLSARCOSINE-AMIDASE-RXN<br><br>ORNDECARBOX-RXN	 STM0701 (*speF*), STM3114 (*speC*)	Putrescine, Spermidine
77	GLYOHMETRANS-RXN<br>PSERTRANSAM-RXN	STM2555 (*glyA*)<br>STM0977 (*serC*)	Phosphatidylethanolamine, Ser, Trp, Phosphatidylserin
78	6PGLUCONOLACT-RXN<br>2TRANSKETO-RXN	STM0785 (*ybhE*)<br>STM2340 (transketolase), STM2341 (transketolase), STM2474 (*tktB*), STM3076 (*tktA*)	nadh, Mthf, Succoa, nad, Coa, AcCoA, His, nadp, UDP-Glucose, dATP, fad, Spermidine, nadph, gtp, Phe, amp, utp, Tyr, ctp, atp, Trp, dCTP, lps, dGTP
79	PPENTOMUT-RXN<br><br>ADENYL-KIN-RXN	STM4569 (*deoB*)<br>STM0488 (*adk*)	nadh, Mthf, Succoa, nad, Coa, AcCoA, His, Asn, nadp, UDP-Glucose, dATP, fad, Cys, Spermidine, nadph, gtp, amp, utp, ctp, atp, Trp, dCTP, Met, dGTP
80	ACETYLGLUTKIN-RXN<br>CREATININASE-RXN	STM4122 (*argB*)	Putrescine, Spermidine
81	6PFRUCTPHOS-RXN<br><br>TRIOSEPISOMERIZATION-RXN	STM1326 (*pfkB*), STM4062 (*pfkA*)<br>STM4081 (*tpiA*)	nadh, nad, Phosphatidylethanolamine, nadp, Phosphatidylglycerol, nadph, DGR, Phosphatidate, clpn, Phosphatidylserin

82	HISTOLDEHYD-RXN<br><br>RXN-8001	STM2072 (*hisD*)<br>STM2072 (*hisD*)	His
83	DIHYDROPICRED-RXN-(NAD)<br>DIHYDROPICRED-RXN-(NADP)	STM0064 (*dapB*)<br>STM0064 (*dapB*)	Peptidoglycan, Lys
84	N-ACETYLGLUTPREDUCT-RXN<br>CREATINASE-RXN	STM4121 (*argC*)	Putrescine, Spermidine
85	RXN0-2382<br><br>TRYPSYN-RXN	STM1726 (*trpA*), STM1727 (*trpB*)<br>STM1726 (*trpA*), STM1727 (*trpB*)	Trp
86	N-ACETYLGLUTPREDUCT-RXN<br>arginine_tx	STM4121 (*argC*)	Putrescine, Arg, Spermidine
87	ACETYLORNTRANSAM-RXN<br><br>CREATININASE-RXN	STM3468 (*argD*)	Putrescine, Spermidine
88	CARBPSYN-RXN<br>CARBAMATE-KINASE-RXN	STM0066 (*carA*), STM0067 (*carB*)<br>STM4466 (carbamate kinase), STM0532 (*argC*)	UDP-Glucose, utp, dTTP, ctp, dCTP
89	ACETYLGLUTKIN-RXN<br>arginine_tx	STM4122 (*argB*)	Putrescine, Arg, Spermidine
90	3.5.2.14-RXN<br><br>N-ACETYLGLUTPREDUCT-RXN	 STM4121 (*argC*)	Putrescine, Spermidine
91	ACETYLORNTRANSAM-RXN<br><br>N-CARBAMOYLSARCOSINE-AMIDASE-RXN	STM3468 (*argD*)	Putrescine, Spermidine
92	HOMOSERKIN-RXN<br><br>THREODEHYD-RXN	STM0003 (*thrB*)<br>STM3708 (*thd*)	Ile, Thr
93	1TRANSKETO-RXN<br><br><br>RIB5PISOM-RXN	STM2340 (transketolase), STM2341 (transketolase), STM2474 (*tktB*), STM3076 (*tktA*)<br>STM3063 (*rpiA*), STM1933 (hypothetical protein)	nadh, Mthf, Succoa, nad, Coa, AcCoA, His, nadp, UDP-Glucose, dATP, fad, Spermidine, nadph, gtp, amp, utp, ctp, atp, Trp, dCTP, dGTP

94	RIBULP3EPIM-RXN<br><br><br>2TRANSKETO-RXN	STM1617 (epimerase), STM4080 (epimerase), STM3483 (*rpe*)<br>STM2340 (transketolase), STM2341 (transketolase), STM2474 (*tktB*), STM3076 (*tktA*)	Mthf, Phe, Tyr, Trp, lps
95	2TRANSKETO-RXN<br><br>GLU6PDEHYDROG-RXN	STM2340 (transketolase), STM2341 (transketolase), STM2474 (*tktB*), STM3076 (*tktA*)<br>STM1886 (*zwf*)	nadh, Mthf, Succoa, nad, Coa, AcCoA, His, nadp, UDP-Glucose, dATP, fad, Spermidine, nadph, gtp, Phe, amp, utp, Tyr, ctp, atp, Trp, dCTP, lps, dGTP
96	O2_tx<br>Glc_tx		nadh, Mthf, Ile, Succoa, nad, Phosphatidylethanolamine, Coa, AcCoA, Star, His, Asn, nadp, Peptidoglycan, Val, UDP-Glucose, Phosphatidylglycerol, dATP, fad, Cys, Thr, Spermidine, nadph, gtp, Phe, amp, DGR, utp, dTTP, Phosphatidate, Lys, Tyr, ctp, atp, Ser, Trp, clpn, Phosphatidylserin, dCTP, Met, Leu, lps, dGTP
97	ACETYLORNTRANSAM-RXN<br><br>arginine_tx	STM3468 (*argD*)	Putrescine, Arg, Spermidine
98	ACETYLORNTRANSAM-RXN<br><br>CREATININE-DEAMINASE-RXN	STM3468 (*argD*)<br>STM3334 (cytosine deaminase)	Putrescine, Spermidine
99	ACETYLGLUTKIN-RXN<br>N-CARBAMOYLSARCOSINE-AMIDASE-RXN	STM4122 (*argB*)	Putrescine, Spermidine
100	SPONTPRO-RXN<br><br>proline_tx		Pro
101	INORGPYROPHOSPHAT-RXN<br><br>ADENPRIBOSYLTRAN-RXN	STM4414 (*ppa*)<br>STM0483 (*apt*)	nadh, Mthf, Succoa, nad, Phosphatidylethanolamine, Coa, AcCoA, Star, His, Asn, nadp, Peptidoglycan, UDP-Glucose, Phosphatidylglycerol, dATP, fad, Cys, Spermidine, nadph, gtp, amp, utp, ctp, atp, Trp, clpn,

			Phosphatidylserin, dCTP, Met, lps, dGTP
102	1.5.1.20-RXN-(NAD)<br><br>1.5.1.20-RXN-(NADP)	STM4105 (*metF*)<br>STM4105 (*metF*)	Mthf, Spermidine, Met

Analysis was conducted using simulated medium composed of glucose, sulphate, phosphate, glycine, alanine, proline, glutamine, arginine, aspartate, glutamate.

Abbreviations (obvious metabolites such as NAD-species are omitted): Mthf – 5-methyl-tetrahydrofolate, His – Histidine, Trp – Tryptophan, Phe – Phenylalanine, lps – Lipopolysaccharide, Tyr – Tyrosine, DGR – 1,2-diacylglycerol, Met – Methionine, clpn – Cardiolipin, Arg – Arginine, Lys – Lysine, Ile – Isoleucine, Thr – Threonine, Asn – Asparagine, Succoa – Succinate CoA, AcCoA – Acetyl-CoA, Star – starch, Val – Valine, Cys – Cysteine, Ser – Serine, Leu – Leucine
